# Fabrication of Ti_2_SnC-MAX Phase Blended PES Membranes with Improved Hydrophilicity and Antifouling Properties for Oil/Water Separation

**DOI:** 10.3390/molecules27248914

**Published:** 2022-12-15

**Authors:** Mahdie Safarpour, Shahla Hosseinpour, Mahsa Haddad Irani-nezhad, Yasin Orooji, Alireza Khataee

**Affiliations:** 1 Department of Chemistry, Faculty of Basic Science, Azarbaijan Shahid Madani University, Tabriz 53714-161, Iran; 2 Research Laboratory of Advanced Water and Wastewater Treatment Processes, Department of Applied Chemistry, Faculty of Chemistry, University of Tabriz, Tabriz 51666-16471, Iran; 3 College of Geography and Environmental Sciences, Zhejiang Normal University, Jinhua 321004, China; 4 Peoples’ Friendship University of Russia (RUDN University), 6 Miklukho-Maklaya Street, Moscow 117198, Russian Federation

**Keywords:** Ti_2_SnC, MAX phase, blended membrane, ultrafiltration, oil separation, reactive sintering

## Abstract

In this research work, the Ti_2_SnC MAX phase (MP) was synthesized via the reactive sintering procedure. The layered and crystalline structure of this MP was verified by SEM, HRTEM, and XRD analyses. This nano-additive was used for improvement of different features of the polyethersulfone (PES) polymeric membranes. The blended membranes containing diverse quantities of the MP (0–1 wt%) were fabricated by a non-solvent-induced phase inversion method. The asymmetric structure of the membranes with small holes in the top layer and coarse finger-like holes and macro-voids in the sublayer was observed by applying SEM analysis. The improvement of the membrane’s hydrophilicity was verified via reducing the contact angle of the membranes from 63.38° to 49.77° (for bare and optimum membranes, respectively). Additionally, in the presence of 0.5 wt% MP, the pure water flux increased from 286 h to 355 L/m^2^ h. The average roughness of this membrane increased in comparison with the bare membrane, which shows the increase in the filtration-available area. The high separation efficiency of the oil/water emulsion (80%) with an improved flux recovery ratio of 65% was illustrated by the optimum blended membrane.

## 1. Introduction

With the development of modern industry, the use of oils and petrochemical products continues to increase, resulting in a massive amount of oily wastewater. This is a major threat to human health and the ecological environment [1]. Due to the presence of insoluble oils, heavy metal ions, and diverse soluble organic pollutants, oily wastewater components are very complicated [2,3]. So, a focus in wastewater treatment is the treatment of wastewater containing multiple pollutants. Because of membrane technology’s low energy consumption, easy operation, and high efficiency, it is among the techniques used in the treatment of oily wastewater [4,5]. However, polymer-based membranes, which are the most used commercial membranes, are facing fouling problems. This is due to their intrinsic hydrophobicity, which limits their performance [6]. There are diverse strategies to decrease fouling of the membranes such as nanomaterial incorporation [7,8] and modification with highly hydrophilic polymers [9,10]. Excellent improvement in membrane stability, antifouling, and hydrophilicity properties is achieved by incorporating hydrophilic materials into the matrix of the polymeric membranes [11,12]. In order to prevent a decrease in membrane separation efficiency, the preparation of blended membranes via physical blending should be avoided, as they can be leached out or agglomerated [13]. Towards this aim, in many studies, different nanoparticles and nanotubes have been utilized. However, composite materials have better performance in membrane fabrication due to the more negative surface charge and large surface area [14]. Among these materials are graphene [15], graphene oxide (GO) [16], transition metal dichalcogenides (TMDs) [17], zeolite [18], and metal-organic frameworks (MOFs) [19]. Despite the aforementioned membranes’ excellent selectivity and permeability, there are some limitations to their performance. For example, the surface of GO is full of carboxyl groups, which become negatively charged by hydration. So, the electrostatic repulsion between GO nanosheets causes the disintegration of the stacked GO membranes in water [20]. Additionally, the exfoliation conditions of MOFs or zeolites are rough, which prevents their application in membrane separation [1]. Hence, new nanomaterial-based membranes with superior properties are needed.

MAX phases (MP) with hexagonal crystalline structures are generally illustrated with the formula of M_n+1_AX_n_, where M, A, and X stand for an early transition metal, an element from the IIIA or IVA groups, and carbon and/or nitrogen, respectively [21]. They are a new group of nanolaminates, which have attracted growing attention due to their inimitable properties. These materials have both metal and ceramic properties, including corrosion resistance, excellent electrical and thermal conductivity, and a great elastic modulus [22]. Via varying elemental composition, the features of MPs can be modified. So, it is expected that their potential will be practicable in different areas soon [23]. Xu et al. [24] synthesized 2D-Ti_3_C_2_T_x_ MXene nanosheets and fabricated Ti_3_C_2_T_x_/chitosan blended membranes. They reported that the presence of the nanosheets does not affect the surface sorption of the membranes while increasing the permeability of the membranes by creating interlayer channels. In another work, Pandey et al. [25] reported the fabrication of cellulose acetate membranes containing 2D-Ti_3_C_2_T_x_ MXene nanosheets. According to their results, these membranes demonstrate good permeability and rejection of dye solutions. The strong M–X bond in the MP has a mixed metallic, covalent, and ionic character, while the M-A bond has a metallic character [26]. Hence, due to the very strong interlayer bonds in the MP, it is difficult to break and convert the MP to MXenes [27].

Here, the reactive sintering method was utilized to synthesize the Ti_2_SnC MP. X-ray diffraction (XRD), field-emission scanning electron microscopy (FESEM), and high-resolution transmission electron microscopy (HRTEM) were used to characterize the synthesized MP morphologically and structurally. Then, the performance of the membranes containing the MP was studied. In this way, the membranes’ flux, hydrophilicity, antifouling properties, and oil/water separation efficiency were probed. According to the literature, there is no report on the use of the Ti_2_SnC MP for improvement of polyethersulfone (PES) polymeric membranes’ performance.

## 2. Results and Discussion

### 2.1. Ti_2_SnC MP Characterization

**Figure 1:**
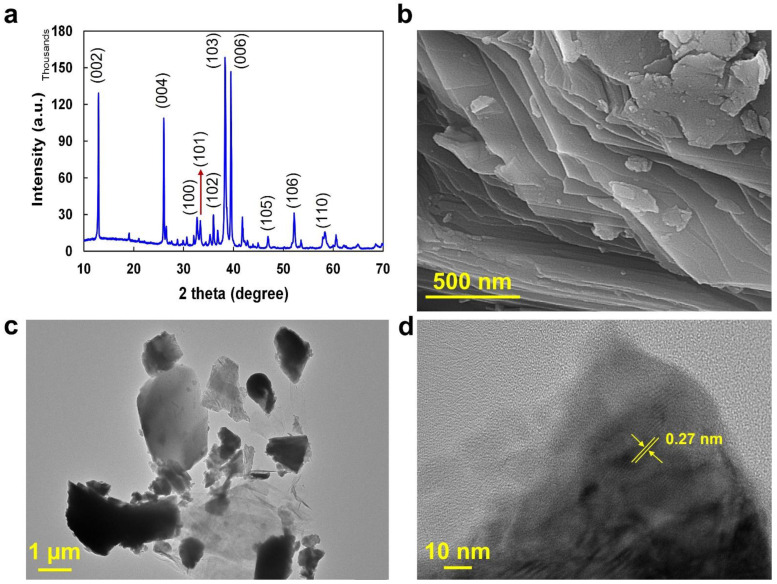
(**a**) XRD pattern, (**b**) SEM, (**c**) TEM, and (**d**) HRTEM micrographs of the Ti_2_SnC MP.

The successful synthesis of the Ti_2_SnC MP was verified via characterization analyses such as XRD, SEM, and TEM. The XRD pattern is shown in Figure 1a, as XRD was performed to evaluate the MP’s crystalline structure. The illustrated peaks at 2 thetas of 12.9, 26.0, 32.7, 33.4, 35.9, 38.3, 39.5, 46.9, 52.1, and 58.3 can be attributed to (002), (004), (100), (101), (102), (103), (006), (105), (106), and (110) reflection planes, respectively [28].

The synthesized MP’s morphology was appraised by FESEM analysis. The micrographs are shown in Figure 1b. According to the images, the MP is composed of compact layers, similar to other MP-based materials that have been reported [28,29]. As shown in Figure 1c, HRTEM images of the MP also demonstrated its layered morphology. Particularly, the lattice with d spaces of 0.27 nm is attributed to the (101) plane of the Ti_2_SnC MP (Figure 1d) [28].

### 2.2. Characterization of Membranes

**Figure 2:**
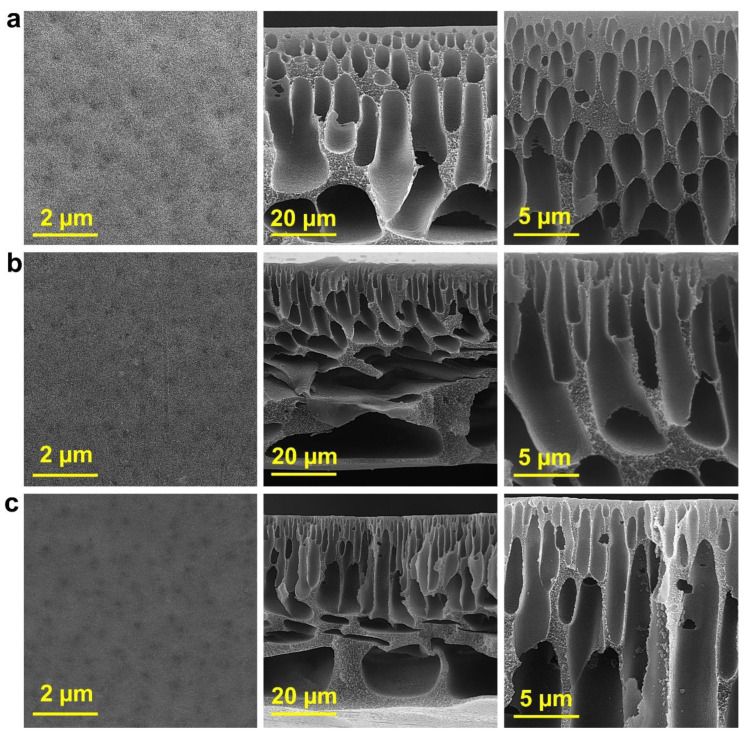

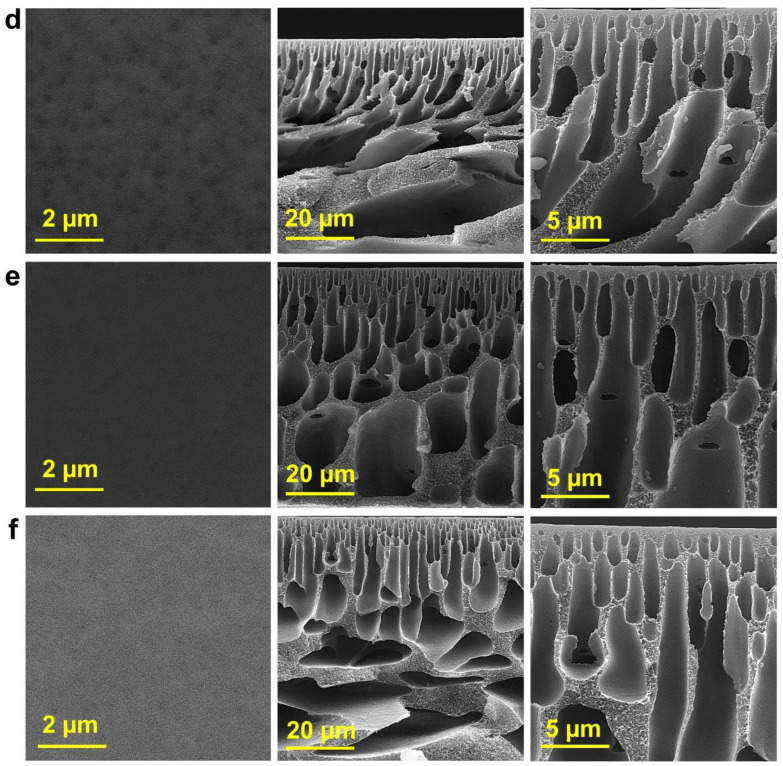
SEM micrographs from surface and cross-sections of various membranes at three different scales of 2, 5, and 20 µm: (**a**) M0, (**b**) M1, (**c**) M2, (**d**) M3, (**e**) M4, and (**f**) M5.

The SEM micrographs from the top surfaces and cross-sections of the different prepared membranes shown in Figure 2. All the blended membranes illustrate the uniformly flat surface without agglomeration, a result of the MP’s homogeneous dispersion in the dope solution. According to the SEM micrographs, the top layer and the sublayer of the membranes are composed of mini voids and coarse finger-like macropores, respectively. The asymmetric structure of the membranes can result from the SEM micrographs. As the cross-sectional morphology of all the blended membranes is the same, the morphology of the membranes was affected by the addition of the MP [30].

**Figure 3:**
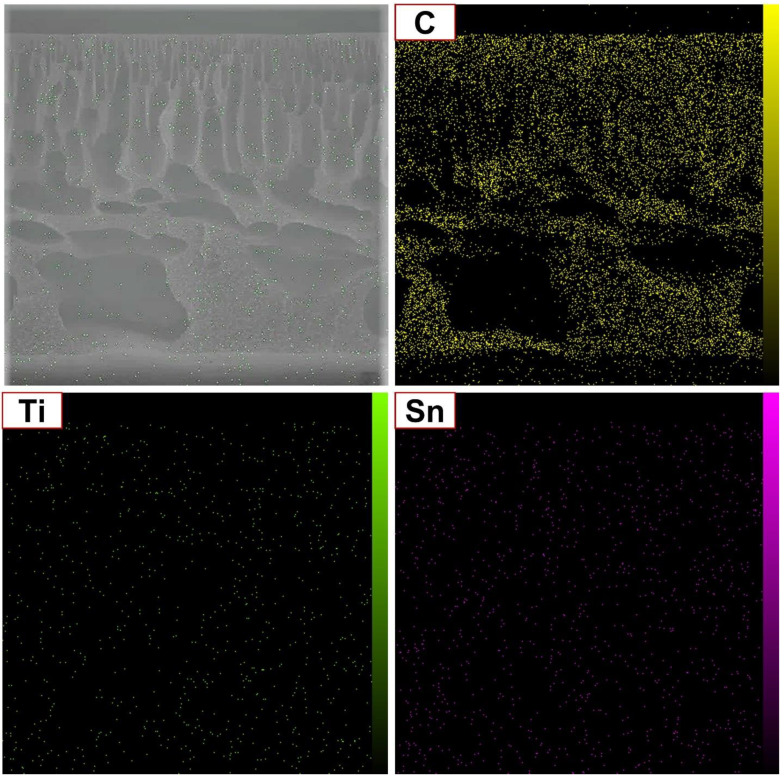
EDAX dot mapping analysis of M3 membrane sample.

To study the dispersal status of the MP in the matrix of the PES membranes, EDAX dot mapping was utilized (Figure 3). Vivid points are dispersed equally in the blended membrane EDAX dot mapping, which shows the homogeneous dispersion of the MP in the matrix of the membranes without any agglomeration. As shown in the EDAX Ti (as indicant of MP) dot mapping, the MP was dispersed in the whole matrix of the membranes. This analysis shows the presence of C, Ti, and Sn in the whole matrix of the membrane homogeneously.

**Figure 4:**
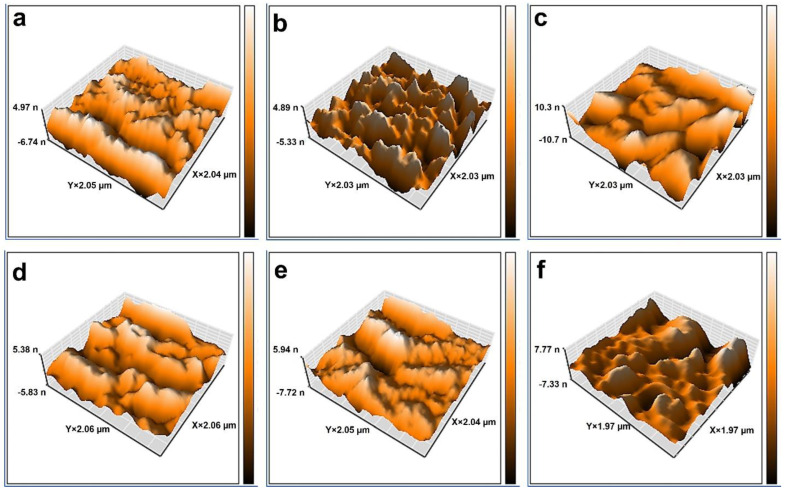
AFM topographical illustrations of various membranes: (**a**) M0, (**b**) M1, (**c**) M2, (**d**) M3, (**e**) M4, and (**f**) M5.

The surface morphology of various blended membranes was probed using AFM analysis. Figure 4 displays the 2 µm × 2 µm 3D AFM images of the membranes. As shown in Table 1, the average surface roughness of the prepared membranes increased via increasing the MP quantity up to 0.5 wt%. The improvement in the permeability of the blended membranes is achieved by high surface roughness, which is related to the efficacious surface increase in filtration [31]. According to the results, the average surface roughness decreased in the membrane containing the MP at over 0.5 wt%, which as a result of the increase in the viscosity of the polymer solution. Moreover, this event caused the formation of membranes with smooth surfaces and condensed structures [32].

Another membrane characterization is contact angle analysis, which is accomplished to probe the membranes’ hydrophilicity. The results show that the hydrophilicity of the membranes is affected by MP quantity. The membrane’s hydrophilicity is affiliated with the roughness of the surface and hydrophilic groups. According to the Wenzel and Cassie model [33,34], in the hydrophilic and hydrophobic matrix, high surface roughness contributes to great hydrophilicity and hydrophobicity properties, respectively. Some hydroxyl groups are formed in the terminable parts of the MP, so, the hydrophilicity of the membrane is improved by incorporating the MP. The improvement in the membrane’s hydrophilicity property is confirmed by the reduced contact angle from 63.38° to 49.77° for M0 and M3, respectively (Table 1). For the last two samples, though the quantity of the hydrophilic group increased, the contact angle increased to 53.28°, hence the hydrophobic property of the membranes dominated in these membranes. Due to the decrease in the membranes’ surface roughness, these membranes show more hydrophobic properties than others [35].

The effect of MP quantity on the blended membranes’ porosity and mean pore size is shown in Table 1. The results demonstrate that the porosity and mean pore size of M0 are lower than that of the others. The polymeric solution containing hydrophilic MP shows an unstable thermodynamic property. Hence, as a result of this phenomenon, solvent and non-solvent exchange occurs quickly, which causes the generation of more holes [36]. M0′s porosity and mean pore size were calculated as 77.69% and 9.27 nm, respectively. By incorporating 0.5 wt% of the MP, these parameters increased to 85.23% and 10.15 nm, respectively. However, there was a decrease in porosity and mean pore size for M4 and M5. Different membranes containing various additives with similar trends have been reported [37,38]. The thermodynamic and rheology features prevail at the lower and greater amounts of 0.5% MP, respectively. On increasing MP quantity above 0.5 wt%, the polymeric solution shows an increase in viscosity, and the rheology feature prevails. High viscosity results in slowing down the de-mixing process, and a condensed structure with low porosity and a small mean pore size is achieved [39].

### 2.3. Performance of the Membranes

#### 2.3.1. Permeability and Antifouling Features of the Membranes

**Figure 5:**
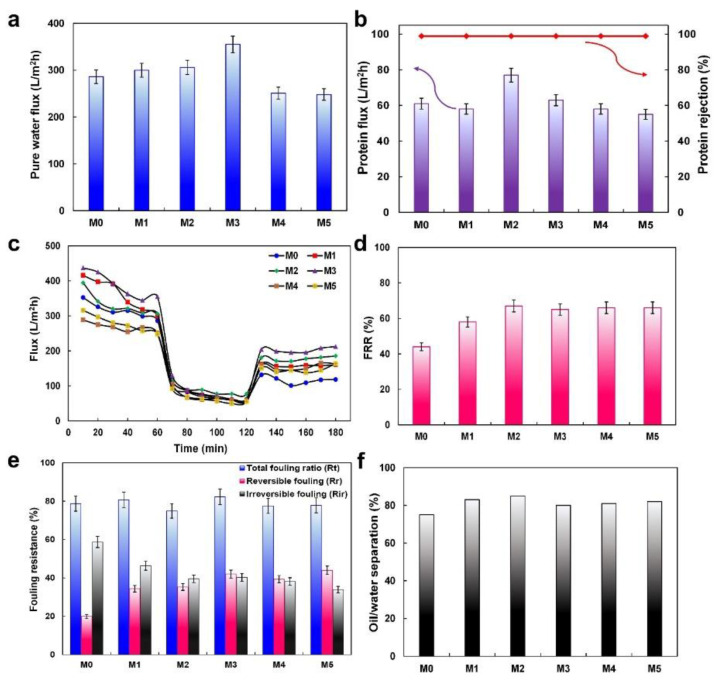
(**a**) The PWF, (**b**) flux and rejection of protein, (**c**) membrane flux against time. “The 1st, 2nd, and 3rd 60 min illustrate the PWF, protein solution flux, and the PWF after membrane cleaning, respectively”. (**d**) FRR%, (**e**) fouling ratio parameters, and (**f**) oil/water separation of different membranes.

The effect of MP quantity on the membranes’ pure water flux (PWF) is demonstrated in Figure 5a. Under operational conditions (60 min filtration, 0.3 MPa), the PWF of the membranes was improved from 286 to 355 L/m^2^ h for M0 and M3, respectively. However, the PWF decreased to 251 and 248 L/m^2^ h for M4 and M5, respectively. Additionally, 0.5 wt% MP causes an increase in protein solution flux from 61 to 63 L/m^2^ h after one-hour filtration (Figure 5b). The reduced fluxes of water and protein for M4 and M5 are related to the blended membrane properties such as hydrophilicity, mean pore size, porosity, and average surface roughness. As mentioned before, 0.5 wt% MP improved the modified membranes’ porosity, mean pore size, and hydrophilicity, which resulted in the facilitation of gravitation and transition of water molecules across the membrane, thus the increase in permeance of the blended membranes. Moreover, the increase in the surface area of the membranes containing the MP increases the permeability. According to the results of the AFM analysis, the average roughness of the membranes increased up to M3, which shows the increase in the filtration-available area. The polymeric solution containing great quantities of the MP had a high viscosity that causes membranes with low porosity, a mean pore size, and average surface roughness. These factors led to the reduction in the PWF and protein solution flux (Table 1).

The antifouling property of the prepared membranes was studied in terms of the flux recovery ratio (FRR), and the results are shown in Figure 5c,d. An improvement in the FRR was seen for all the modified membranes (67.0%) compared with the bare membrane (44.0%). According to the results, adding the MP in the PES membrane’s matrix results in an improvement of the antifouling properties. Thus, protein molecules are more easily removed from the surface of the modified membranes by washing compared to from the bare membrane. When these results coordinate with the blended membranes’ contact angle, it can be concluded that a thin water layer forms by the absorption of water molecules on the hydrophilic surface of the blended membranes, which acts as a barrier and avoids the adsorption of foulants [40]. In order to investigate the fouling resistance of the membranes, total (R_t_), reversible (R_r_), and irreversible (R_ir_) resistances, three parameters of the fouling, were measured (Figure 5e). R_r_ and R_ir_ occur as a result of weak and strong interactions between the foulant and the surface of the membrane. Water rinsing can revive R_r_, but the membrane will be damaged by R_ir_. As shown in Figure 5e, M0 showed the lowest R_r_ (19.9%) and the highest R_ir_ (58.7%), with increased and decreased values in the modified membranes, respectively. The obtained R_r_ and R_ir_ were 42.0% and 40.3%, respectively, for M3. The hydrophilic properties of the MP might be the reason for the improved antifouling properties of the fabricated membranes. The available hydroxyl groups are increased by the MPs in the membranes’ matrix. These groups, via constituting hydrogen bonds, lead to a thin water layer film forming simply on the surface of the membranes. This film prevents molecules from adhering, thus the antifouling feature of the membranes improves.

#### 2.3.2. Membrane Separation Ability

With the aim of appraising the separation efficiency, the filtration of an oil/water emulsion was carried out for 1 h. As shown in Figure 5f, the efficiency of separation was 75% and ≥80% for the bare and modified membranes, respectively. As described before, the attachment of oil droplets on the surface was reduced by the formation of the hydration layer on the membranes’ surface [41]. Another oil removal factor in the ultrafiltration processes is the size exclusion, so, by increasing the filtration time, the removal efficiency is increased. At the first, the blended membranes’ pores are completely open, and the oil droplets can easily transfer across the membrane. With time, some pores are blocked via the oil particles, thus the concentration of oil decreases in the permeate, and oil rejection efficiency is increased [42]. Finally, a comparison table between the performance of the 0.5 wt% MP blended membrane and other reported membranes was prepared (Table 2).

## 3. Experimental

### 3.1. Chemicals

Titanium and tin (Sn) powders with a purity of 99.99% and 1000 mesh were purchased from STNMT Co. Ltd. (Shandong, China). Aladdin Reagent Co. Ltd. (Shanghai, China) was the supplier that graphite powder (99.99%) was acquired. PES was prepared from Ultrason E6020P (Ludwigshafen, Germany). Polyvinylpyrrolidone (PVP) was procured from Rahavard Tamin (Tehran, Iran). N, N-Dimethylacetamide (DMAc) was purchased from Merck (Tokyo, Japan). Bovine serum albumin (BSA) was prepared from Optimum Nutrition (Dublin, Ireland). Motor oil was purchased from Castrol (Tehran, Iran).

### 3.2. Ti_2_SnC MP Synthesis

The Ti_2_SnC MP was synthesized by the customized reactive sintering procedure inspired by the literature [48,49]. Towards this aim, a revolution speed of 350 rpm in the planetary ball mill apparatus (ball:material = 10:1) was utilized to blend the powders of tin, graphite, and titanium with a molar ratio of Ti:Sn:C = 2:1.2:1 for 12 h. In order to compress the blended powder into a green part, a pressure of 250 MPa was applied. Then, this green compact part was placed in an argon-gas-powered tube furnace with an airflow rate of 100 SCCM. The sample was subjected to a sintering proceeding in which it was heated to 1200 °C with non-linear steps and heating rate of 1–10 °C/min. The synthesized powder was obtained when the sample was cooled, grinded, sieved and the purity increased by acid wash [28].

### 3.3. MP/PES Blended Membranes Fabrication

In order to fabricate the MP/PES blended membranes, a phase inversion procedure was applied [50]. First, a stock solution of the MP was prepared and dispersed using an ultrasonic bath (60 min) to be utilized for preparing suspensions of the MP with various concentrations. In this way, different quantities of the MP suspension (0, 0.1, 0.2, 0.5, 0.75, and 1 wt%) were dispersed in the DMAc and sonicated for 90 min. Then, after 1 h of adding PVP (1 wt%), PES (18 wt%) was added to the abovementioned suspension and stirred vigorously for 24 h. The obtained homogeneous solutions were degassed via placing them in an oven with a temperature of 55 °C for 4 h. The polymeric films were cast on clean and dry glass plates, and the films’ thickness was 150 µm. Tap water was used as the non-solvent to submerge the casted films at room temperature. Distilled water was used to maintain the obtained membranes for further use. Different blended membranes were identified by “M0, M1, M2, M3, M4, and M5” for membranes containing “0, 0.1, 0.2, 0.5, 0.75, and 1 wt% MP”, respectively.

### 3.4. Characterization Techniques

Different characteristic methods were applied to identify the synthesized Ti_2_SnC MP, which can be pointed to HRTEM (JEM-2100 Plus electron microscope, JEOL, Akishima, Japan), XRD (SmartLab, Akishima, Japan, using Cu Ka radiation at 40 kV and 100 mA), EDX, and FESEM (Tescan Mira3 microscope, Brno, Czech Republic, accelerating voltage: 15 kV). Studying the dispersion of the MP in the membrane matrix was performed using EDX dot mapping. Contact angle analysis was carried out to probe the membranes’ surface hydrophilicity (the reported result was an average of 3 detections). Pollutant concentrations (protein and oil) were detected by using a UV–Vis spectrophotometer and chemical oxygen demand (COD) analysis, respectively. The membranes’ mean pore radius (rm) and overall porosity (ε) were determined by the Guerout–Elford–Ferry equation and the gravimetric analysis, respectively [51]. The gravimetric analysis is introduced as Equation (1):

(1) $$\varepsilon =\frac{W_{1\;}{-\;W}_{2}}{A\;\times \;l\;\times \;d_{w}}$$

where d_w_ (0.998 g/cm^3^), A (cm^2^), l (cm), W_1_ (g), and W_2_ (g) represent the density of water, the effective area of the membrane, the membrane’s thickness, and the wet and dry weight of the membrane, respectively.

Equation (2) describes the Guerout–Elford–Ferry equation, in which ∆P, Q, l, η, A, and ε are related to transmembrane pressure (3 × 10^5^ Pa), permeated pure water per unit time (m^3^/s), the membrane’s thickness (m), water viscosity (8.9 × 10^−4^ Pa.s), the membrane’s effective surface area (m^2^), and overall porosity, respectively.

(2) $$r_{m}=\sqrt{\frac{\left(2.9-1.75\;\varepsilon \right)\times 8\eta \mathrm{lQ}}{\varepsilon \times A\;\times \Delta P}}$$

### 3.5. Membranes’ Filtration Procedure

The filtration tests of the membranes were carried out using a dead-end filtration apparatus. First, a pressure of 0.4 MPa was used to pre-compact the membranes through the filtration of distilled water for 20 min. Then, the filtration of distilled water (J_W,1_), protein (500 mg/L, J_P_), and distilled water (J_W,2_) was accomplished to probe the flux and FRR properties of the membranes at a pressure of 0.3 MPa for 60 min, respectively. In order to omit the formation of a cake layer and reversible fouling of the membranes after the filtration of protein, they were washed out and plunged into distilled water for 20 min.

## 4. Conclusions

In this research work, the Ti_2_SnC MP, with high hydrophilic properties, was used as an additive to improve the features of the PES membrane. The characterization analyses approved the successful synthesis of the MP by a reactive sintering procedure. Different blended membranes containing diverse quantities of the MP (0–1 wt%) were fabricated through the phase inversion technique. As a result of the homogeneous dispersion of the MP in the dope solution, membranes with uniform flat surfaces without any agglomeration were obtained. The asymmetric structure of the membranes, with small and finger-like holes, does not change in the bare and modified membranes, so the mechanism of the membrane formation does not change as a result of the presence of the additive in the dope solution. The average surface roughness, mean pore size, hydrophilicity, and porosity increased on adding 0.5 wt% MP. With 0.5 wt% MP in the matrix of the PES membrane, the flux and the FRR increased from 286 to 355 L/m^2^ h and 44% to 65%, respectively. The modified membranes separated the oil/water emulsion by over 80%, compared to by 75% by the bare membrane. According to the results, this membrane can be useful for oily wastewater purification.

## Figures and Tables

**Table 1:** Surface roughness, contact angle, porosity, and mean pore size of the prepared membranes.

Membrane	Porosity (%)	Mean Pore Size (nm)	Contact Angle (°)	Roughness Parameters		
				S_a_ (nm)	S_q_ (nm)	S_y_ (nm)
M0	77.6 ± 1.4	9.27 ± 0.97	63.3 ± 2.9	6.29	8.12	40.02
M1	79.7 ± 1.6	9.71 ± 0.19	54.1 ± 6.7	7.32	8.95	47.30
M2	83.1 ± 1.3	9.72 ± 0.33	50.7 ± 2.5	8.70	11.65	100.01
M3	85.2 ± 5.6	10.14 ± 0.69	49.7 ± 4.6	8.82	10.80	62.92
M4	84.3 ± 2.9	8.41 ± 0.30	51.5 ± 1.8	8.43	10.56	46.16
M5	79.6 ± 3.5	8.15 ± 0.80	53.2 ± 1.9	8.17	11.46	55.35

**Table 2:** Collation of the efficiency of the 0.5 wt% Ti_2_SnC MP/PES membrane with other PES-based blended membranes in the literature.

Membrane	Nano-Additive	Pore Former	Operational Pressure (MPa)	Pure Water Flux (L/m^2^h)	FRR (%)	Ref.
PES (20 wt.%)	GO (0.3 wt.%)	T904 (5 wt.%)	0.1	245	62	[43]
PES (20 wt.%)	MWCNT (0.1 wt.%)	PVP (1 wt.%)	0.5	23	95	[44]
PES (17.25 wt.%)	A-PCC (3 wt.%)	PEG (1.75 wt.%)	0.15	180	86.4	[45]
PES (11.54 wt.%)	HMO (0.75)–TiO_2_ (0.25)	PVP (1.15 wt.%)	0.1	28.48	91.5	[46]
PES (18 wt.%)	PANI/Fe_3_O_4_ (0.1 wt.%)	PVP (1 wt.%)	0.4	52	80	[47]
PES (18 wt.%)	Ti_2_SnC MAX phase (0.5 wt.%)	PVP (1 wt.%)	0.3	355	65	This work

## Data Availability

The data presented in this study are available on request from the corresponding author.

## Sample Availability

Samples of the compounds are not available from the authors.
